# Phytases from *Enterobacter* and *Serratia* species with desirable characteristics for food and feed applications

**DOI:** 10.1007/s13205-016-0378-x

**Published:** 2016-02-13

**Authors:** Harpreet Kaur Kalsi, Rajveer Singh, Harcharan Singh Dhaliwal, Vinod Kumar

**Affiliations:** Department of Biotechnology, Akal College of Agriculture, Eternal University, Baru Sahib, Sirmour, 173101 India

**Keywords:** Phytase, Phytate, Micronutrient bioavailability, Phosphorus, Monogastric animals

## Abstract

Phytases are enzymes of great industrial importance with wide range of applications in animal and human nutrition. These catalyze the hydrolysis of phosphomonoester bonds in phytate, thereby releasing lower forms of *myo*-inositol phosphates and inorganic phosphate. Addition of phytase to plant-based foods can improve its nutritional value and increase mineral bioavailability by decreasing nutritional effect of phytate. In the present investigation, 43 phytase positive bacteria on PSM plates were isolated from different sources and characterized for phytase activity. On the basis of phytase activity and zone of hydrolysis, two bacterial isolates (PSB-15 and PSB-45) were selected for further characterization studies, i.e., pH and temperature optima and stability, kinetic properties and effect of modulators. The phytases from both isolates were optimally active at the pH value from 3 to 8 and in the temperature range of 50–70 °C. Further, the stability of isolates was good in the pH range of 3.0–8.0. Much variation was observed in temperature and storage stability, responses of phytases to metal ions and modulators. The *K*_m_ and *V*_max_ values for PSB-15 phytase were 0.48 mM and 0.157 μM/min, while for PSB-45 these were 1.25 mM and 0.140 μM/min, respectively. Based on 16S rDNA gene sequence, the isolates were identified as *Serratia* sp. PSB-15 (GenBank Accession No. KR133277) and *Enterobacter cloacae* strain PSB-45 (GenBank Accession No. KR133282). The novel phytases from these isolates have multiple characteristics of high thermostability and good phytase activity at desirable range of pH and temperature for their efficient use in food and feed to facilitate hydrolysis of phytate-metal ion complex and in turn, increased bioavailability of important metal ions to monogastric animals.

## Introduction

Most of the cereals and legumes are rich in carbohydrates and proteins but some of them have certain antinutritional factors which restrict their use in food. One such antinutritional factor is the phytic acid (*myo*-inositol 1,2,3,4,5,6-hexa*kis*phosphate; IP_6_) which is the major storage form of phosphorus (60–80 %), in soil (Turner et al. 2002), grains (Lott et al. 2000) and manures from monogastric animals (Barnett 1994). It acts as an antinutrient as it chelates various micronutrients such as Ca^2+^, Fe^2+^, and Zn^2+^ and diminishes their bioavailability in digestive tract of monogastric animals due to lack of enzyme for hydrolysis of phytate complexes (Kumar et al. 2014, 2015). Therefore, consumption of food containing large amount of phytate may cause severe mineral deficiency. The micronutrient deficiency could be alleviated using exogenous phytase addition (Kumar et al. 2015), development of phytase secreting transgenic plants (Brinch-Pedersen et al. 2000), and production of low phytate mutant plants to use in food and feed of the humans (Raboy 2001). Addition of phytase during food processing is most preferred as the other two approaches may lead to decreased seed phytate leading to deleterious effect on germination and decreased agricultural field. Phytases (*myo*-inositol hexaphophohydrolase; EC. 3.1.3.) are the class of phosphatases which are capable of hydrolysing phytic acid with the release of inorganic phosphorus and divalent cations. So far, many phytases from microbes have been demonstrated to be useful as feed additive in monogastric animals and various phytase preparations are commercially available. None of the commercial phytases investigated, satisfied all of the criteria of an ideal phytase for use in animal feed (Boyce and Walsh 2006). Therefore, most of the research on phytases has been focused on isolation and characterization of novel phytases from different sources (Choi et al. 2001; Greiner and Farouk 2007; Huang et al. 2009; Kumar et al. 2014; Lei et al. 2007). The present study, therefore, was carried out to isolate and screen rhizospheric soil bacterial isolates for extracellular phytase production with suitable biochemical properties and use in food and feed.

## Materials and methods

### Chemicals and reagents

All the chemicals and reagents used in this study were of molecular biology and analytical grade and procured from standard manufacturers as GeNei, Sigma, Merck and HiMedia Pvt. Ltd.

### Isolation, screening and selection of phytate solubilizing bacterial isolates

#### Isolation of phytate hydrolyzing bacteria

Phytate solubilizing bacteria were isolated from different sources (poultry farm, rhizospheric soils, compost and degraded wood samples) taken from Baru Sahib, Sirmour, Himachal Pradesh (Latitude 30.8244, longitude 77.26855). The isolation of bacterial isolates was achieved using serial dilution approach as described in Kumar et al. (2013). 100 µl samples from the final dilution were plated directly onto phytase screening media (PSM; 10 g l^−1^ d-glucose, 4 g l^−1^ Ca-phytate, 2 g l^−1^ NH_4_NO_3_, 0.5 g l^−1^ KCl, 0.5 g l^−1^ MgSO_4_·7H_2_O, 0.01 g l^−1^ FeSO_4_·7H_2_O, 0.01 g l^−1^ MnSO_4_·H_2_O, 15 g l^−1^ Agar) agar plates and incubated at 37 °C for 24–48 h (Kerovuo et al. 1998). The isolates from all sources forming hydrolysis zones were purified and further screened for their phytate hydrolysis potential. The plates were then examined for halo zone around bacterial culture and solubilization Index (SI) was calculated as, SI = (colony diameter + halo zone diameter)/colony diameter (Premono et al. 1996). Pure cultures of phytase positive bacterial isolates were maintained on LB agar plates and stored as stab cultures as well as glycerol stocks. The isolates were given name as PSB (phytase secreting bacteria) followed by numerals.

#### Production of phytase enzyme

To determine and further select potential isolates based on their phytase production ability, the isolates were grown in LB media overnight and 1 % inoculum (0.5 ml) of this primary culture was added to 25 ml of sterile phytase production media (PSM;10 g l^−1^ d-glucose, 4 g l^−1^ Na-phytate, 2 g l^−1^ NH_4_NO_3_, 0.5 g l^−1^ KCl, 0.5 g l^−1^ MgSO_4_·7H_2_O, 0.01 g l^−1^ FeSO_4_·7H_2_O, 0.01 g l^−1^ MnSO_4_·H_2_O) in conical flasks as described by Kerovuo et al. (1998). The culture was incubated in incubator shaker at 37 °C with 200 rpm for checking phytase production at different time intervals (24, 48 and 72 h). Cell debris at these time intervals was removed by centrifugation at 10000 rpm for 15 min, 4 °C. The supernatant was kept in fresh tube and taken for estimation of extracellular phytase activity.

#### Estimation of phytase activity

Phytase activity was determined by following the standard method of (Engelen et al. 1993). The reaction mixture contained 100 µl of enzyme sample and 900 µl of 0.1 M acetate buffer, pH 6.0. It was followed by addition of 500 µl of 5 mM sodium phytate (prepared in 0.1 M acetate buffer, pH 6.0) as substrate. The reaction was carried out at 37 °C for 30 min, and then stopped by adding 500 µl freshly prepared colour reagent. In blank reaction, colour reagent was added prior to the incubation and substrate solution (added after incubation). The colour developed from the phytase activity was determined at 415 nm. One unit phytase was defined as the amount of enzyme that released 1 µM of inorganic phosphate in 1 min. The amount of phosphorus released was calculated based on standard curve of KH_2_PO_4_.

### Identification of finally selected PSB isolates

The 16S rDNA sequencing using universal primers (518F: 5′-CCAGCAGCCGCGGTAATACG-3′ and 800R: 5′-TACCAGGGTATCTAATCC-3′) were used for species level identification of selected PSB isolates. The pure culture maintained on stab culture and glycerol stocks were sent for custom sequencing service from Macrogen USA. The amplified PCR products were sequenced by automated DNA sequencer (96-Capillary ABI DNA sequencer). The obtained sequences were analysed using BlastN with existing sequences in NCBI database and then deposited in NCBI GenBank (http://www.ncbi.nlm.nih.gov/GenBank/submit.html). The evolutionary history was inferred using the Neigbour-Joining method (Saitou and Nei 1987). Evolutionary analyses were conducted by MEGA5 (Tamura et al. 2011).

### Biochemical and kinetic characterization of phytase enzyme from selected isolates

Based on halo zone formation and maximum phytase activity at different time, phytase enzyme from two isolates (PSB-15 and PSB-45) was selected for characterization of their kinetic properties, i.e., pH optimum, temperature optimum, *K*_m_ and *V*_max_. As reported, the enzymes are affected by metal ions and some other modulators; effect of some metal ions and modulators was also estimated on concentrated phytase extracted from these isolates.

#### Partial purification of crude phytase using ammonium sulphate fractionation and dialysis

Crude phytase samples were partially purified using precipitation with 70 % ammonium sulphate at 4 °C. The precipitate was again dissolved in 2 ml 0.1 M acetate buffer (pH-6.0) and dialyzed against 0.1 M acetate buffer (pH-6.0) with 3–4 times change of buffer solution to remove salt from the concentrated enzyme solution. The partially purified/concentrated samples were assayed for phytase activity, protein content and kinetic characterization of phytase enzymes from these selected isolates.

#### Effect of pH on enzyme activity and stability

The optimum pH for the activity of the PSB isolates was determined by standard phytase assay with following buffers (0.1 M); glycine–HCl (pH 2.0–3.0), sodium acetate buffer (pH 4.0–6.0) and Tris–HCl buffer (pH 7.0–9.0). Maximum enzyme activity was taken as 100 % and relative activity in other was calculated. Graphs were plotted between pH vs enzyme activity (% relative activity).

To determine pH stability of PSB phytates, 350 µl of the enzyme was preincubated with 350 µl of different buffers such as 0.1 M glycine–HCl buffer (pH 2.0–3.0), 0.1 M acetate buffer (pH 4.0, 5.0, 6.0 and 6.5), 0.1 M Tris–HCl (pH 7.0 and 9.0) at 4 °C for 2 h, in absence of phytic acid. After incubation, phytase activity was estimated in incubated samples using standard phytase assay with sodium acetate buffer. The residual enzyme activity was calculated. Graph was plotted between pH vs  % residual activity.

#### Effect of temperature on enzyme activity and stability

The optimum temperature for phytase activity of PSB isolates was determined by performing the routine phytase assay at different temperatures, i.e., 30, 40, 50, 60, 70 and 80 °C. The enzyme activity was calculated as % relative activity in comparison to maximum activity obtained at a particular temperature.

For estimation of thermostability, enzyme in acetate buffer (0.1 M, pH 6.0) was incubated at 70, 80 and 90 °C for different time intervals (10 min and 20 min). After incubation, 500 µl of sodium phytate substrate solution (5 mM, prepared in 0.1 M Acetate Buffer pH 6.0) was added in each test tube. Reaction was carried out at 37 °C for 30 min and the residual activity of the enzyme was calculated as per standard protocol. Graph was plotted between incubation time vs % residual activity.

#### Effect of metal ions on enzyme activity

The ionic solutions of metals (5 mM), i.e., Cu^2+^, Mg^2+^, Mn^2+^ and Fe^2+^ were prepared in acetate buffer (0.1 M, pH 6.0). 50 µl enzyme in acetate buffer (0.1 M, pH 6.0) was preincubated with 500 µl of different metal ions at 37 °C for 30 min. After incubation, 950 µl acetate buffer followed by 500 µl sodium phytate solution (5 mM, pH 6.0) was added to the reaction mixture. The reaction mixture was again incubated at 37 °C for 30 min. Reaction was stopped by adding 500 µl colour stop reagent. The residual enzyme activity was measured at λ_415_ nm. The residual activity was calculated and plotted against respective metal ions.

#### Effect of modulators on enzyme activity

The effects of enzyme modulators {ascorbic acid, ethylenediamine-tetraacetic acid (EDTA), *β*-mercaptoethanol, DTT and urea at a final concentration of 5 mM} on the PSB phytase activity was measured in the reaction mixture as described ahead. Enzyme solution (100 µl) was pre-incubated with 900 µl acetate buffer with respective modulator for 30 min at 37 °C, followed by the standard enzyme assay as described earlier. The activity assayed in the absence of the modulators was taken as the control.

#### Determination of *K*_m_ and *V*_max_

To determine Michaelis–menten constant *K*_m_, phytase activity was determined at different concentration of sodium phytate (0.25–10.0 mM) in acetate buffer (0.1 M, pH 6.0). The reaction mixture was prepared and phytase activity was determined as described earlier. The prepared reaction mixture was incubated at 37 °C for 30 min and OD was taken at λ_415_ nm. The *K*_m_ and *V*_max_ values were determined using the Lineweaver–Burk double reciprocal plot (Lineweaver and Burk 1934).

### Growth, biochemical and morphological characteristics of selected isolates

The selected PSB isolates were tested for their ability to grow on varying pH (5–11) and salt concentration (0–10 % NaCl). Gram staining was performed using HiMedia Gram Stain Kit following manufacturer’s instructions. Motility test for selected isolates was also performed using LB agar media in test tubes. Briefly, PSB isolate’s culture was streaked on top of autoclaved media in test tubes. Growth of colony inside media along with top was an indication of bacterial motility. The colony morphology (shape, texture, margins and pigmentation) of selected isolates was observed to easy identification and maintaining pure culture of the isolates.

### Digestion efficiency of PSB phytases

Digestion efficiency of phytase extractions from selected PSB isolates was determined on soybean meal. The experimental reaction mixture in test tubes contained 1 g of defatted soybean meal suspended in 5 ml of acetate buffer (0.1 M, pH 6.0) in each tube. PSB phytases was added in tubes in the ratio of 0 (no phytase), 0.25 U of phytase (i.e., 0, 250 U/kg, respectively). Tube without added PSB phytase was considered as control. The mixture was incubated at 37 °C for 2 h prior to the determination of inorganic phosphate contents in each tube. Increase in inorganic phosphorus content on addition of phytase extract in tubes was an indication of efficient phytate hydrolysis in soybean meal and good digestion efficiency. Phosphorus content was determined according to (Chen et al. 1956). The amount of phosphate released was calculated based on standard curve of KH_2_PO_4_.

## Results

### Isolation, screening and selection of phytase producing bacterial isolates

**Fig. 1:**
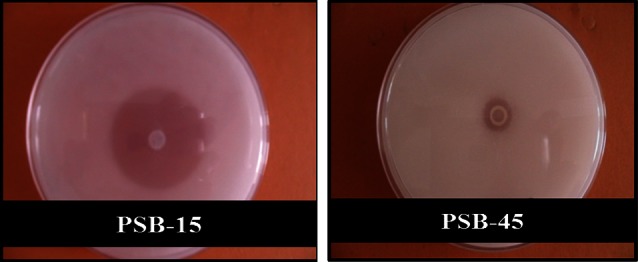
Selected PSB isolates produced zone of clearance on PSM plates with Ca-phytate

In the present study, bacterial strains from different sources (Poultry farm soil, Rhizospheric soils, Compost and Degraded wood samples taken from Baru Sahib, Himachal Pradesh, India) directly isolated and screened on PSM plates. Initially, randomly picked 43 isolates forming zone of hydrolysis were purified and again grown on PSM plates to reconfirm their phytase production ability (Fig. 1). All the 43 bacterial isolates were confirmed as phytase secreting bacteria (PSB). Selection was made on the basis of halo zone formation. Highest number of PSB isolates (9 isolates) were obtained from compost (PSB-37, PSB-38, PSB-39, PSB-40, PSB-41, PSB-42, PSB-43, PSB-44, PSB-45) followed by rhizospheric soil from millet (8 isolates; PSB-4, PSB-5, PSB-6, PSB-7, PSB-8, PSB-9, PSB-10, PSB-11), poultry farm soil (8 isolates; PSB-17, PSB-18, PSB-19, PSB-20, PSB-21, PSB-22, PSB-23, PSB-24) and degraded wood (7 isolates; PSB-27, PSB-28, PSB-29, PSB-30, PSB-31, PSB-32, PSB-33) samples. Soil samples from other sources like rhizospheric soil from soybean and maize also resulted into 4 and 3 isolates, respectively. The PSB isolates along with their isolation source are given in Table 1.

**Table 1:** PSB isolates from different sources

S. no.	Isolation source	PSB isolates
1	Maize rhizospheric soil	PSB-1, PSB-2, PSB-3
2	Millet rhizospheric soil	PSB-4, PSB-5, PSB-6, PSB-7, PSB-8, PSB-9, PSB-10, PSB-11
3	Soybean rhizospheric soil	PSB-13, PSB-14, PSB-15, PSB-16
4	Poultry farm soil	PSB-17, PSB-18, PSB-19, PSB-20, PSB-21, PSB-22, PSB-23, PSB-24
5	Soil from hill	PSB-25, PSB-26
6	Wood degraded (porous wood from hills)	PSB-27, PSB-28, PSB-29, PSB-30, PSB-31, PSB-32, PSB-33
7	Plant roots (hills)	PSB-35, PSB-36
8	Compost (Eternal University compost plant)	PSB-37, PSB-38, PSB-39, PSB-40, PSB-41, PSB-42, PSB-43, PSB-44, PSB-45

**Fig. 2:**
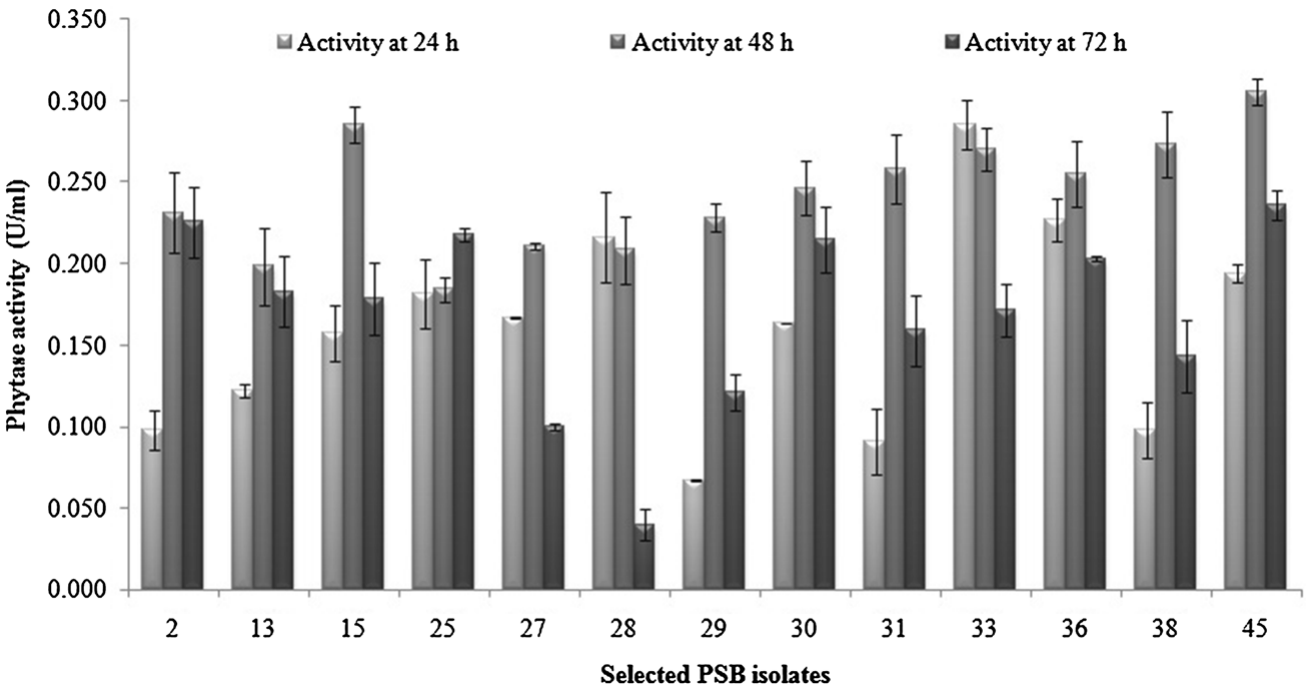
Comparative phytase activity of selected PSB isolates at different time

To make further selection and find out best phytase producing PSB isolates after selection of PSB isolates on the basis of formation of halo zone on calcium phytate plates (Fig. 1), these were assayed for phytase activity (U/ml) in liquid PSM media under standard conditions at different time intervals, i.e., 24, 48 and 72 h. Results presented in Fig. 2 revealed different isolates had varying phytase activity under given conditions at different time periods. Some PSB isolates had maximum phytase activity at 24 h, but most of the cultures possessed significantly good range of activity at 48 h and subsequent decrease on 72 h of incubation, except a few. Different trends were obtained with phytase activity vs time of incubation and depending on isolate and its source of isolation. The enzyme activity varied from 0.006 to 0.305 U/ml depending on the above factors. It was interesting to notice that more than 60 %, i.e., 16 out of 26 isolates from soils (10 out of 16 from rhizospheric soils) and 6 out of 9 isolates from compost sample produced maximum phytase activity after 72 h of incubation. On the other hand, more than 85 % of isolates (6 of 7 isolates) from wood samples produced maximum phytase activity before 72 h (at 24 and 48 h) of incubation under given conditions of growth. Different PSB phytases showed different enzyme activity in PSM. Maximum phytase activity at 48 h was shown by PSB-45 (0.305 U/ml) followed by PSB-15, PSB-33, PSB-38 and PSB-31 (0.285, 0.285, 0.273 and 0.258 U/ml, respectively) (Fig. 2).

Based on zone of hydrolysis and highest phytase activity, isolates PSB-15 and PSB-45 were selected for further biochemical characterization studies. To determine the phytate solubilization index, the isolates were further grown on PSM plates supplemented calcium phytate by point inoculation. The isolates were found to produce clearance zone of varying diameter on phytase screening medium containing calcium phytate as substrate. Among these, PSB-15 showed maximum diameter of zone of 3.4. Diameter of zone by PSB-45 was only 0.6 cm. These values suggested that PSB-15 had higher ability to use phytate as substrate at 37 °C as compared to other PSB isolates. The solubilization index of PSB-15 and PSB-45 were 35 and 7, respectively.

### 16S rDNA-based identification of selected bacterial isolates

**Fig. 3:**
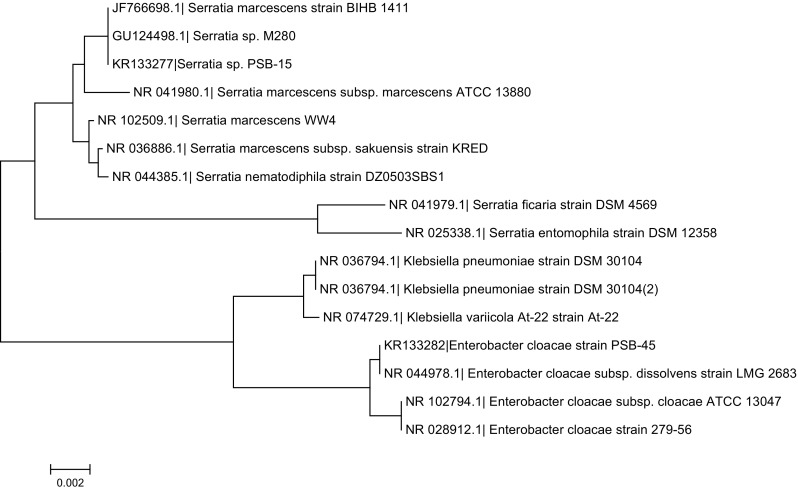
Phylogenetic relationships of PSB isolates with related taxa by Neighbour-Joining method based on 16S rRNA gene sequences

Considering phytase production potential and desirable biochemical characteristics (thermostability, pH stability, pH and temperature optima) of phytases from selected isolates, the isolates PSB-15 and PSB-45 were identified as *Serratia* sp. PSB-15 (GenBank Accession No. KR133277) and *Enterobactor cloacae* strain PSB-45 (GenBank Accession No. KR133282), based on analysis of 16S rDNA gene sequence. The sequences showed 99 % similarity with existing sequence in NCBI database. The evolutionary history was inferred using the Neighbuor-Joining method. The optimal tree with the sum of branch length = 0.05866441 is shown in Fig. 3. The tree is drawn to scale, with branch lengths in the same units as those of the evolutionary distances used to infer the phylogenetic tree.

### Biochemical and kinetic properties of phytase from selected isolates

**Fig. 4:**
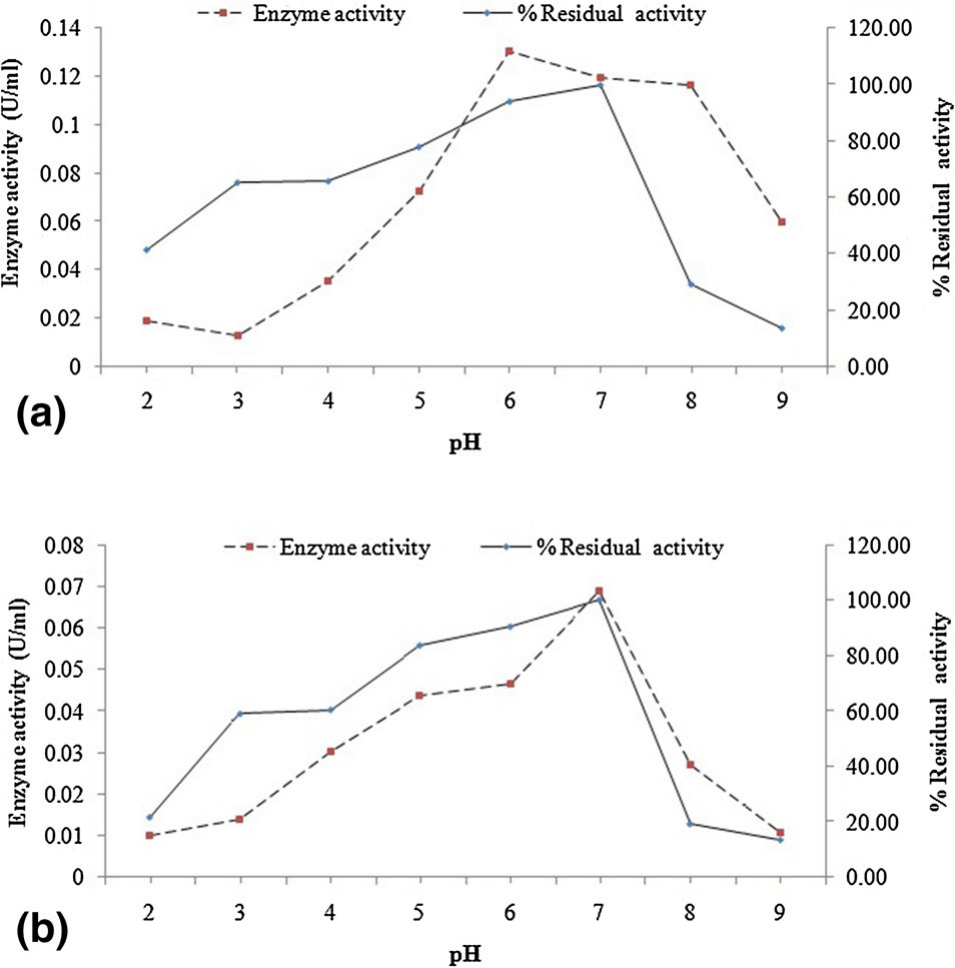
Effect of different pH on activity and stability of **a** PSB-15 phytase and **b** PSB-45 phytase

The use of ammonium sulphate fractionation (70 %) and dialysis methods resulted into purification fold and yield of 2.18 and 44.63 % for PSB-15 phytase, and 2.55 and 62.30 for PSB-45 phytase, respectively. These phytase enzymes from the selected isolates were further characterized for optimum pH and temperature for their maximum activity. For pH optimum, enzyme activity was estimated using different pH buffers (pH 2.0–9.0). Both of these isolates gave maximum phytase activity in the pH range of 5.0–8.0 (Fig. 4a, b). Phytases from PSB-15 and PSB-45 were maximum active at pH 6.0 and 7.0, respectively.

**Fig. 5:**
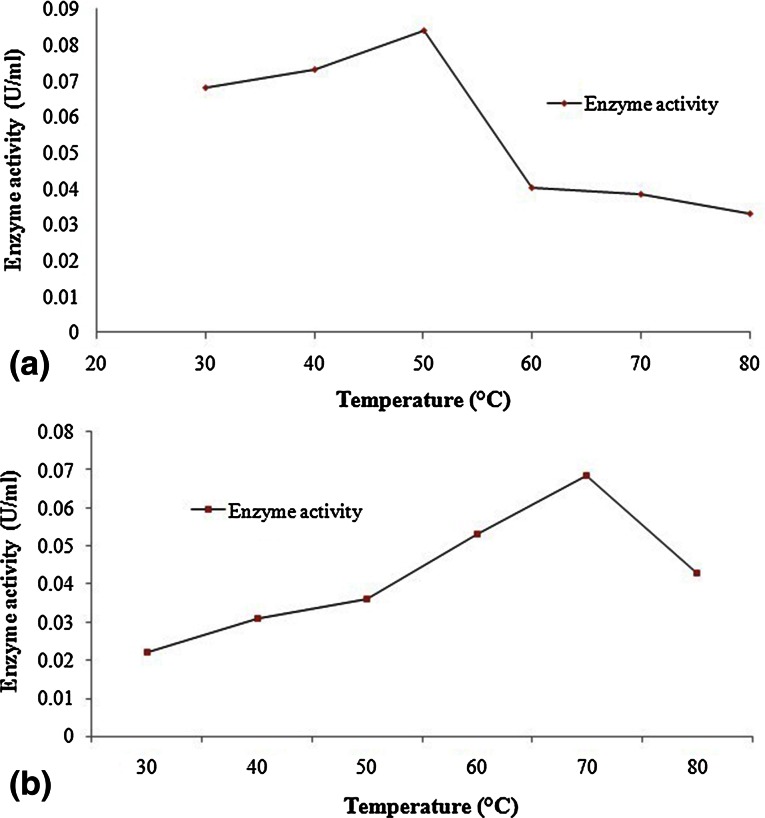
Effect of different temperature on activity of **a** PSB-15 phytase and **b** PSB-45 phytase

The optimum temperature for maximum phytase activity was varied from 50 to 70 °C under given conditions of enzyme activity estimation (Fig. 5a, b). Phytases from PSB-15 isolate was most active at 50 °C while PSB-45 isolate had maximum phytase activity at 70 °C and it was more thermophilic phytase from compost sample.

**Fig. 6:**
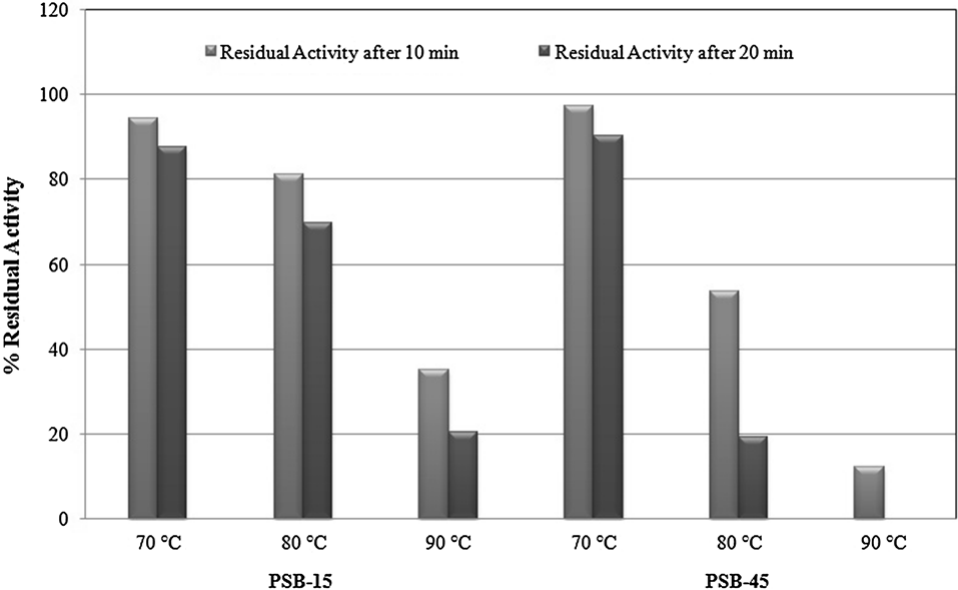
Thermostability of PSB phytases at different temperatures

Phytase enzymes from selected isolates were highly stable in broad pH range (pH 3–7 for PSB-15 and pH 3–8 for PSB-45) as revealed in Fig. 4 and Table 2. The thermal stability of phytase and the ability of the heat-denatured enzyme to refold were investigated at 70, 80 and 90 °C. After treating at 80 °C for 10 min, PSB-15 still retains 81.1 % of its initial activity, followed by 69.8 % activity after treatment at same temperature for 20 min. It was more stable than PSB-45 phytase which showed only 52.2 and 18.3 % residual activity after treatment at the same temperature for 10 and 20 min, respectively (Fig. 6).

**Table 2:** PSB isolation sources, phytase production characteristics and properties of phytases from selected PSB Isolates

S. no.	Characteristic features	PSB isolates	
		PSB-15	PSB-45
Culture characteristics			
1	Sources of PSB isolate	Soybean rhizospheric soil	Compost
2	Maximum enzyme production time (h)	48	48
3	Maximum enzyme activity (U/ml) in PSM	0.285	0.305
4	Change in pH of production media after 72 h (Control-6.7)	3.3	3.4
5	Phosphorus solubilization index	35	7
Enzyme characteristics			
5	Optimum pH	6.0	7.0
6	Optimum temperature	50 °C	70 °C
7	pH stability	3-7	3-8
8	Stability after 30 d at 4 °C/RT (% residual activity as compared to fresh enzyme)	96.5/5.6	45.5/35.8
9	*K*_m_ (mM)	1.25	0.48
10	*V*_max_ (μM/min)	0.157	0.140

Investigation of the storage stability of phytases after 30 days revealed variation in stability (% residual activity) with storage temperature and PSB isolate. Analysis of results for storage stability for 30 days at room temperature (RT) in Table 2 revealed that PSB-45 phytase had stability of 35.8 % even at RT. PSB-15 phytase was highly unstable to store for 30 days at RT as only 5.6 % residual phytase activity was reported. In contrast to this, analysis of storage stability for 30 d at 4 °C revealed that very good stability (96.5 % residual activity of its initial activity) was shown by PSB-15 phytase over PSB-45 phytase (45.5 % residual activity).

To look upon whether the change in pH also affects the enzyme activity with time, pH value of each production medium was tested at 72 h and compared with the control (pH of fresh production media). In both cases, a decrease in pH was observed to 3.3 and 3.4 for PSB-15 and PSB-45, respectively, as compared to 6.7 of control (Table 2).

### Effect of metal ions and modulators on phytase activity

In this study, sulphate ions of different metals (Fe, Cu, Mn and Mg) were used to check activity of phytases in the presence of these metal ions (Table 3). Ferrous sulphate, copper sulphate, manganese sulphate, magnesium sulphate were used for the study. Different metals inhibit enzyme activity to different extent and phytase from both PSB isolate behaved differently to each metal ion. The relative activity profile with ferrous sulphate was about 50 % for PSB-15 phytase while for PSB-45 phytase it was only 35.8 %. With copper sulphate PSB-15 was stable and showed 78 % relative activity while PSB-45 phytase was least stable with copper sulphate among all the studied metal ions. Manganese sulphate also showed more inhibition in case of PSB-45 phytase. Magnesium sulphate also inhibited enzyme activity more than 50 % in both cases.

**Table 3:** Effect of metal ions (5 mM concentration) on phytase activity (% relative activity as compared to control) of selected PSB isolates

S. no.	Modulator/metal ion	% relative activity	
		PSB-15 Phytase	PSB-45 Phytase
1	Control	100	100
2	Ferrous sulphate	49.9	35.8
3	Copper sulphate	78.7	26.2
4	Manganese sulphate	72.6	36.6
5	Magnesium sulphate	38.9	46.2
6	Mercaptoethanol	33.3	74.6
7	Urea	41.9	17.9
8	Ascorbic acid	52.8	37.3
9	DTT	67.6	54.3
10	EDTA	35.4	70.8

Effect of several modulators (mercaptoethanol, urea, ascorbic acid, DTT and EDTA) on activity of phytases from selected PSB isolates was examined and represented as % relative activity as compared to control enzyme in absence of these modulators (Table 3). On incubation with these modulators, different PSB phytase behaved differently. It was inferred that mercaptoethanol and EDTA were most effective in reducing the phytase activity of PSB-15 (~65 % inhibition) while DTT was least effective. In case of PSB-45 urea inhibited phytase activity more than 80 % while mercaptoethanol and EDTA were least effective (>70 % relative activity) in reducing the phytase activity.

### Effect of substrate concentration on phytase activity and determination of *K*_m_ and *V*_max_

Effect of varying concentration of phytate on phytase activity from selected PSB isolates was estimated and is given in Table 2. The *K*_m_ values for PSB-15 and PSB-45 phytases were 1.25 and 0.48 mM, respectively. The *V*_max_ value of phytase from PSB-15 and PSB-45 were 0.157 and 0.140 U/ml, respectively.

### Growth, biochemical and morphological characteristics of selected PSB isolates

Characteristics of PSB isolates including various parameters related to morphological and biochemical tests are given in Table 4. To determine growth characteristics, the growth of PSB isolates at various pH range and various salt concentration was studied to check pH tolerance and salt tolerance, respectively. As shown in Table 4, selected strains grew well at neutral pH. In addition, PSB-15 grew well from pH 5.0 to 9.0. Gram staining was another parameter considered for characterization of bacterial isolates. Both the studied isolates were gram negative and motile in nature. Other morphological features including colony shape, colour, arrangement and texture were also observed and given in Table 4.

**Table 4:** Growth, biochemical and morphological characteristics of selected PSB Isolates

S. no.	Characteristic feature	PSB isolates	
		PSB-15	PSB-45
Growth characteristics			
1	pH	5–9	5–9
2	Salt concentration	0–2.5	0–5.0
Biochemical characteristics			
3	Starch test	−	+
4	Ammonia test	+	+
5	VRBA test	−	+
6	Citrate test	−	+
7	Gram stain test	−	−
8	Motility test	+	+
Morphological characteristics			
9	Shape of colony	Plane/raised	Plane/raised
10	Margins	Entire	Entire
11	Colour/pigment	Cream	Cream
12	Texture	Wrinkled	Smooth shiny

### Digestion efficiency of PSB phytases

**Fig. 7:**
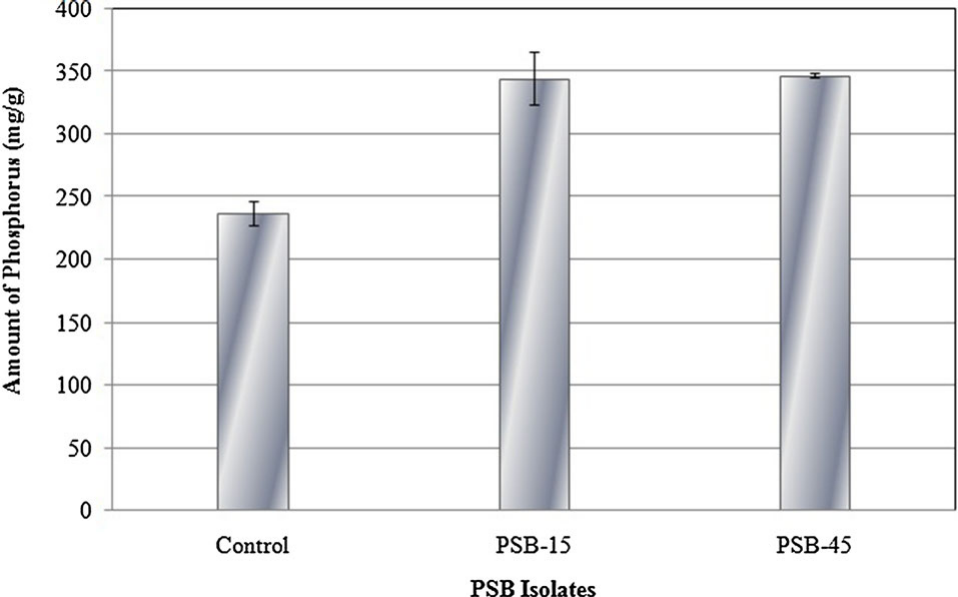
*Bar chart* showing digestion ability of PSB phytases using soybean meal

Considering the above observations, selected phytase were tested for their ability to increase phosphate content defatted soybean meal. For each experiment reaction, use of 250 U/kg of each phytase in incubated feed mixture at 37 °C for 2 h resulted into increased phosphorus in both the cases, corresponding to no addition of enzyme in control. The effect of PSB phytase application on amount of free phosphorus is given in Fig. 7. In comparison to control (feed without phytase), incubation of feed with phytase (250 U/kg) resulted into an increased liberation of phosphate in the range of 45–46 % from soybean meal.

## Discussion

Phytase is an enzyme chemically known as myo-inositol hexakisphosphate phosphohydrolase. It hydrolyzes phytic acid to myo-inositol and phosphoric acid in a stepwise manner forming myo-inositol phosphate intermediates. The addition of microbial phytase has been seen as a way to reduce the level of phosphate pollution in areas of intensive animal production. Numerous studies have shown the effectiveness of supplementation of microbial phytases in improving utilization of phosphate from phytate. Therefore, inorganic phosphorous supplementation in the diets of monogastric animals can be obviated by including adequate amounts of phytase and as a result, the faecal phosphate excretion of these animals may be reduced by up to 50 % (Walz and Pallauf 2002). Moreover, phytate acts as anti-nutrient by binding to protein and chelating minerals. Addition of phytase can improve the nutritional value of plant based foods by enhancing protein digestibility and mineral availability through phytate hydrolysis. Since certain myo-inositol phosphates have been proposed to have novel metabolic effects, phytase may be used to produce functional food. Although phytase can be commercially produced from four commercial phytase preparations viz. NatuphosTM (BASF, *Aspergillus niger* phytase), RonozymeTM (DSM, *Peniophora lycii* phytase), PhyzymeTM (Diversa/Danisco A/S, *Schizosaccharomyces pombe* phytase, and QuantumTM (Diversa/Syngenta, *Escherichia coli* phytase), none of the commercial phytases assessed satisfied all of the criteria of an ideal phytase for use in animal feed. Because phytases with the required properties for animal feed application have not been found in nature so far, studies are ongoing to identify phytases which are more suited to animal feed applications than current commercial preparations.

Though the enzyme is widely distributed in nature, microorganisms are considered potentially most suitable for industrial exploitation due to ease of cultivation and manipulation of cultural conditions in the minimum space and time. Microbial phytases are actively secreted into soil, where they participated both in decomposition of fresh plant debris and in the liberation of phosphorus from soil organic compounds. Thus, microbial phytases are the key enzymes of the P cycling in the soil (Jorquera et al. 2008). The importance of soil microorganisms for increasing the availability of phosphorus from phytates to plant roots has been suggested by Tarafdar and Marschner (Tarafdar and Marschner 1995). Similarly, (Richardson et al. 2000) had also highlighted the potential role of soil microorganisms for increasing the availability of phosphorus from phytates through phytase action. In earlier studies, known phytate-degrading organisms that include aerobic bacteria, anaerobic bacteria and fungi were isolated from different environmental conditions but bacteria as a source of the enzymes are cheaper and take less time for enzyme production than other sources. Survey of microorganisms for selection of potential phytase producers was the subject of study by several workers (Kumar et al. 2013; Yanke et al. 1999; Yoon et al. 1996). Different workers used multiple sources for isolation of phytase positive isolates including different kinds of soils, animals and plants. Wood (rotten) as a source for phytase producing microbes (*Aspergillus niger*) was used by Vats and Banerjee (2002).

In the present study also, although different sources were used for isolation of PSB isolates, degraded wood as source of novel phytase positive isolates also emerged as potential source of these microbes. Degraded wood was reported as a source of tannin degrading *Serratia* sp. by Pepi et al. (2010). Ability of bacterial isolates from varying habitats to produce extracellular phytase reveals their high diversity and further strengthens chances of getting potential isolates for a suitable phytase with desired properties. The phytase from *Serratia* sp. PSB-15 with good phytate solubilization index, extracellular phytase production, thermostability and varying activity parameters was found to be interesting and could be utilized for further application purposes.

The use of calcium phytate as the sole phosphorus and carbon source and formation of halo zone on PSM plates and phytase activity in media was effectively used for selection of phytase secreting/phytate solubilizing bacteria, which has been adopted in several previous studies on *Bacillus amyloliquefaciens* (Kerovuo et al. 1998), *Bacillus sp.* DS11 (Kim et al. 1998) and *B. subtilis* strains (Shimizu 1992), where each produced a positive response to the plate clearing as well as an enzyme activity assay. It is proved to be a useful and effective strategy for such studies on screening and isolation of phytase positive isolates as growth on plates is usually easier than in a liquid medium for phytate-degrading bacteria (Choi et al. 2001; Richardson et al. 2005), hence no further assessment need to be conducted on isolates that could not grow or form halo zone on PSM plates. Further, no direct correlation was found between halo zone on PSM plates to the phytase activity in liquid media which might be due to different specificity of PSB phytases for sodium phytate and calcium phytate as in liquid media sodium phytate was used compared to calcium phytate in PSM plates. Some strains produced clearance zone but the diameter of the clearance zone and growth of the organism were comparatively very slow, which could be a reflection of the isolates inability to degrade phytate or perhaps, merely an indication of unfavourable conditions for phytase production.

Decreasing pH during phytase production in liquid culture is a common feature of phosphate solubilizing bacterial isolates as major microbiological means by which insoluble phosphate compounds are mobilized is the production of organic acids, accompanied by acidification of the medium. The organic and inorganic acids convert tricalcium phosphate in soil to di- and mono-basic phosphates with the net result of and enhanced availability of the element to the plants (Song et al. 2005). Decreased media pH during this study revealed their possible application as phosphate solubilizer and liquid bio-fertilizer for increasing plant growth and productivity. Characterization of these PSB isolates for their ability to solubilize different kinds of phosphate compounds will be the very important aspect of further studies on these isolates.

The ability of a phytase to hydrolyze phytate in the digestive tract is determined by its enzymatic properties. As stomach is the main functional site of supplemental phytase, an enzyme with an acidic pH optimum is certainly desirable. The main area of concern for the phytase is the low activity and stability under different conditions of pH. Although, the optimum pH of enzyme activity from both isolates was in the common range of pH 4.5–7.5 for maximum activity of general bacterial phytases viz. *Pseudomonas syringae* MOK1 and *Pseudomonas* sp. (pH 5.5), *Enterobacter* sp. 4 (pH 5.5), *Obesumbacterium proteus* (pH 4.9), *Escherichia coli* (pH 4.5), *Bacillus* sp. DS11 (pH 7.0), *Bacillus* sp. (pH 6.0) and *Bacillus subtilis* (natto) N-77 (6.0-6-5) (Golovan et al. 1999; Kerovuo et al. 1998; Kim et al. 1998; Popanich et al. 2003; Shimizu 1992; Yoon et al. 1996; Zinin et al. 2004), but results revealed these bacterial phytases as desirable with good activity and stability at pH in the range of 3.0–8.0. Activity and stability (with >50 % residual activity) of selected *Serratia* and *Enterobacter* strains was in this range of pH. According to (Mukhametzyanova et al. 2012), bacterial phytases of *Bacillus* and *Enterobacter* species generally have pH optimum in the range from 6.0 to 8.0, but phytase from this study was even stable at lower pH and shown to be higher stable at such pH values and their usability under condition of low pH in stomach. From a physiologically relevant standpoint, these phytases displayed significant activity and stability between pH 3.0 and 6.0, a range necessary to facilitate phytate degradation in mouth (pH 5.0–7.0), stomach (fed state pH 6.5, reducing to 3.5–4.5 upon stimulation of acid secretion) and upper part of the duodenum (pH 4.0–6.0). The loss of activity under high alkaline and acidic conditions might be due to the protein structure of phytase changed under the strong alkali or acid conditions to make phytase lose its activity (Jorquera et al. 2008). The PSB phytases, those showed very good activity at acidic and alkaline pH could be used as feed additive. Both selected PSB phytases show good activity at acidic and alkaline pH.

As per expectations of getting an extremophile organism and thermostable phytase from compost, we have obtained *Enterobacter cloacae* strain PSB-45 phytase with novel properties including high optimum temperature for maximum phytase activity which were differ from other *Enterobacter* sp. phytase as reported by Yoon et al. (1996). Although the enzyme from PSB-45 isolate had high temperature optima but it was not having desired thermostability at higher temperature. The temperature optimum for *Enterobacter cloacae* strain PSB-45 phytase was higher than PSB-15 phytase. Similar temperature optima for maximum phytase activity was reported in *Bacillus* sp. DS11 and *Klebseilla aerobgenes* (Jareonkitmongkol et al. 1997; Kim et al. 1998). Temperature optima for phytase from isolate PSB-15 was higher than that of optimum temperatures reported by other workers, i.e., 40 °C for that from *Bacillus* sp. KHU-10 (Choi et al. 2001), 45 °C for that from *Obesumbacterium proteus* (Zinin et al. 2004), 58 °C for that from *A. Niger* 11T53A9 (Greiner et al. 2009) and comparable to *Bacillus subtilis* (natto) N-77 (Shimizu 1992).

Thermal stability of phytase and the ability to refold to an active conformation are important properties from a commercial perspective. Enzyme thermal stability is pertinent in animal feed applications (Pandey et al. 2001), where the enzyme preparations are normally incorporated into the meals before pelleting. The pelleting of feed helps animals to have a balanced diet and facilitates preservation from feed borne pathogens. However, during the pelleting process the enzyme is exposed to temperatures around 65–95 °C with holding times from 1 to 10 min (Bohn et al. 2007). Therefore, all feed enzymes need to be heat stable to avoid substantial activity loss during this process. Although phytase inclusion using an after-spray apparatus for pelleted diets and/or chemical coating of phytase may help bypass or overcome the heat destruction of the enzyme, phytases resisting high temperatures will no doubt be better candidates for feed supplements. As revealed from results some of the isolates were reported more than 80 % residual phytase activity after such treatment. In this regard, *Serratia* sp. PSB-15 was observed to be best among studied phytase in this study. It exhibited very good tolerance and retained 81 % of its original activity at 80 °C for 10 min and consistently, retained ~70 % activity up to 20 min at 80 °C. It was even more thermostable than commercially available phytases from *A. niger* (retained 50 % of the initial activity for 10 min at 70 °C) and *P. lycii* (completely inactivated after 15 min at 70 °C) (Greiner and Farouk 2007). Garrett et al. (2004) also investigated the thermostability of phytase from *E. coli* and they found that it did not lose any activity after heating for 1 h at 62 °C and retained only 27 % residual activity when heated at 85 °C for 10 min. At temperatures between 50 and 55 °C, it underwent an irreversible conformational change that resulted in 70–80 % loss of enzyme activity. From comparison with above studies, it could be said that phytase enzyme from our source isolates (*Serratia* sp. PSB-15 and *Enterobacter cloacae* strain PSB-45) are suitable for pelleting and their displayed higher thermal stability would be an added advantage for potential commercial interest.

Residual activity of the crude enzyme from both isolates at 4 °C was determined after 30 days. Investigation of the storage stability of PSB phytase after 30 days showed different values. From this study it was found that only isolates PB-15 retained more than 95 % activity. Storage stability data clearly revealed that enzyme from these isolates could not be stored for longer or if needed, alternate strategies must be adopted for long time storage of PSB phytases. When the enzyme was kept at room temperature, phytase activity decreased as compared to when enzyme was stored in refrigerator after 30 days.

There are various reports in which metal ions have been shown to modulate phytate-degrading activity. Phytases from different microbes differed in their requirement for metal ions for their activity. The inhibitory effect of metal ions on phytase activity might be ascribed to the strong chelating ability of phytate, where binding of metal ions on phytate might impair access of the enzyme to the functional group on the phytate molecule leading to decreased activity (Maenz et al. 1999). These results are in conformity with (Kim et al. 1998), who reported that phytase of *Bacillus* sp. DS11 was strongly inhibited by Mn^2+^ and moderately inhibited by Mg^2+^ and Cu^2+^ at 5 mM concentration. Yanke et al. (1999) also reported that the mixtures containing 5 mM Fe^2+^, Cu^2+^, Zn^2+^ and Hg^2+^, strongly inhibited phytase activity of *Selenomonas ruminantium*. The partially purified enzyme from *Klebsiella oxytoca* MO-3 was strongly inhibited by Zn^2+^, Fe^2+^, and Cu^2+^. Presence of Mg^2+^, Mn^2+^ and Cr^2+^ inhibited enzyme activity of r-PhyB49, a BPPhy from *Serratia* sp. (Zhang et al. 2011). Hong et al. (2011) also observed decrease in enzyme activity of *Bacillus* phytase in presence of Mg^2+^, Mn^2+^, Cr^2+^ and Co^2+^. Phytase from *Enterobacter* sp. 4 was inhibited by each addition of 1 mM Zn^2+^, Ba^2+^, Cu^2+^, Al^3+^ and EDTA (Yoon et al. 1996).

The observations from effect of modulators inferred that mercaptoethanol was least effective in reducing the activity of PSB phytases except PSB-25 which was least stable. As reducing reagents 2-mercaptoethanol and dithiotreitol (DTT) have no major effects on PSB phytases suggesting that these enzymes either have no free and accessible sulfhydryl groups play a negligible role in the enzyme activity and structure. In case of urea, it inhibits both PSB phytases activity >50 % suggesting that as concentration of urea increases, the kinetics of inactivation and the denaturation by urea increases linearly (Xiang et al. 2004). In case of ascorbic acid, selected PSB-15 phytases was found to be unstable with ascorbic acid. Ascorbic acid forms protonated chelates by coordinating with metal ions at low intermediate pH, while unprotonated chelates at higher pH (Martell 1982). Presence of EDTA inhibited PSB-15 phytase activity to large extent (35 % relative activity). Inactivation of phytase by EDTA was also reported in phytase from *Bacillus* sp. (Rao et al. 2008) and *B. licheniformis* (Wang et al. 2011). Thus, decreased PSB phytases activity with EDTA might be due to interference in availability of metal ions required for activity. Similar to phytase from *Enterobacter* sp. 4, phytase from *Enterobacter cloacae* strain PSB-45 was inhibited by EDTA (Yoon et al. 1996).

Kinetic parameters (*K*_m_ and *V*_max_) for dephosphorylation of phytate by phytase have been studied widely. The Michalis–Menten constant (*K*_m_) and the maximal reaction velocity (*V*_max_) of the PSB phytases were determined by assay of PSB phytases with sodium phytate in the concentrations range of 0.5–10 mM as the *K*_m_ values of other phytases has been reported in a range of 0.08–10 mM (Nayini 1986). The kinetic constants, *K*_m_ for the PSB-15 and PSB-45 phytases were reported as 0.48 and 1.25 mM, respectively. The *K*_m_ and affinity of *Serratia* sp. PSB-15 phytase toward phytate was in accordance to the *Bacillus subtilis* natto N-77 (*K*_m_ = 0.50 mM), *A. niger* (*K*_m_ = 0.606 mM), *Bacillus laevolacticus* (*K*_m_ = 0.526 mM) (Gulati et al. 2007; Shimizu 1992; Vats and Banerjee 2002). The *K*_m_ of *E. cloacae* strain PSB-45 phytase was comparable to *Kodamaea ohmeri* BG3 phytase (1.28 mM) (Li et al. 2008). The isolates differed in their morphological, growth and biochemical characteristics and these characteristics may be useful while utilizing these isolates for optimization and large scale production of phytase enzyme.

Literature survey suggested that the phytase dose of 250–1500 U/kg feed was considered suitable for proper hydrolysis of phytate complexes in feed and it dependent on several factors such as animal type, phytase sources, diet formulation (amount of substrate for phytase) and selected response parameters (Kumar et al. 2014). Thus, the increased phosphorus up to 45 % over control with the use of 250 phytase U/Kg meal in present study further support the potential applicability of both the phytases in food and feed. A similar increase in phosphorus content was also reported by application of a recombinant phytase by Kumar et al. (2014). It could have a significant impact on the enhanced bioavailability of phosphorus and other phosphorus bound micronutrients into food and feed under consideration.

## Conclusion

In conclusion, this study resulted into characterization of multiple bacterial isolates up to their kinetic properties with some key findings discussed above. Among characterized and finally selected isolates, *Serratia* sp. PSB-15 and *Enterobacter cloacae* PSB-45 have shown multiple characteristics of good thermostability, phytase activity and solubilization index, and desirable range of pH and temperature optima for their potential application in food and feed for increased bioavailability of important minerals.
